# Non-syndromic Oculocutaneous Albinism: Novel Genetic Variants and Clinical Follow Up of a Brazilian Pediatric Cohort

**DOI:** 10.3389/fgene.2020.00397

**Published:** 2020-04-28

**Authors:** Laire Schidlowski, Fernando Liebert, Pérola Grupenmacher Iankilevich, Priscila Regina Orso Rebellato, Rafaela Andrade Rocha, Nadia Aparecida Pereira Almeida, Aayushee Jain, Yiming Wu, Yuval Itan, Roberto Rosati, Carolina Prando

**Affiliations:** ^1^Instituto de Pesquisa Pelé Pequeno Príncipe, Curitiba, Brazil; ^2^Faculdades Pequeno Príncipe, Curitiba, Brazil; ^3^Hospital Pequeno Príncipe, Curitiba, Brazil; ^4^Department of Genetics and Genomic Sciences, Icahn School of Medicine at Mount Sinai, New York, NY, United States; ^5^The Charles Bronfman Institute for Personalized Medicine, Icahn School of Medicine at Mount Sinai, New York, NY, United States

**Keywords:** oculocutaneous albinism, melanogenesis, sequencing, exome, *TYR*, *SLC45A2*

## Abstract

Oculocutaneous albinism (OCA) is a genetic disorder characterized by skin, hair, and eye hypopigmentation due to a reduction or absence of melanin. Clinical manifestations include vision problems and a high susceptibility to skin cancer. In its non-syndromic form, OCA is associated with six genes and one chromosomal region. Because OCA subtypes are not always clinically distinguishable, molecular analysis has become an important tool for classifying types of OCA, which facilitates genetic counseling and can guide the development of new therapies. We studied eight Brazilian individuals aged 1.5–18 years old with clinical diagnosis of OCA. Assessment of ophthalmologic characteristics showed results consistent with albinism, including reduced visual acuity, nystagmus, and loss of stereoscopic vision. We also observed the appearance of the strabismus and changes in static refraction over a 2-year period. Dermatologic evaluation showed that no participants had preneoplastic skin lesions, despite half of the participants reporting insufficient knowledge about skin care in albinism. Whole-exome and Sanger sequencing revealed eight different mutations: six in the *TYR* gene and two in the *SLC45A2* gene, of which one was novel and two were described in a population study but were not previously associated with the OCA phenotype. We performed two ophthalmological evaluations, 2 years apart; and one dermatological evaluation. To the best of our knowledge, this is the first study to perform clinical follow-up and genetic analysis of a Brazilian cohort with albinism. Here, we report three new OCA causing mutations.

## Introduction

Albinism is a group of rare genetic disorders defined by disruption of melanin biosynthesis, resulting in hypopigmentation and severe visual deficits. It is classified into ocular albinism (OA) which is characterized by hypopigmentation of the ocular tissue, and oculocutaneous albinism (OCA) in which lack of pigmentation affects the hair, skin, and eyes (Grønskov and Brondum-Nielsen, 2007; Marti et al., 2018). The worldwide prevalence of albinism is estimated at 1:17,000. Although albinism has worldwide distribution, prevalence varies according to the type of albinism and ethnicity, mainly because of distinct founder mutations in different genes (Montoliu et al., 2014; Federico and Krishnamurthy, 2018; Kromberg, 2018).

Oculocutaneous albinism is a group of autosomal recessive disorders which are classified into seven types according to the affected gene. Mutations in the *TYR* gene are related to OCA subtype 1 (Tomita et al., 1989), which is the most prevalent subtype of albinism among Europeans and Euro-descendents, and is subdivided into OCA1A and OCA1B. Due to the complete absence of tyrosinase activity, individuals with OCA1A present blue to pink irises and white hair and skin. Therefore, OCA1A is the most severe form of OCA. Except for OCA1A, OCA1B, and the other OCA subtypes develop some pigmentation over their lifetime. Individuals with OCA1B develop some pigmentation over their lifetime (Grønskov and Brondum-Nielsen, 2007; King and Summers, 2010). Mutations in *OCA2*, *TYRP1*, *SLC45A2*, *SLC24A5*, and *LRMDA* have been attributed to subtypes OCA2, OCA3, OCA4, OCA6, and OCA7, respectively (Rinchik et al., 1993; Boissy et al., 1996; Newton et al., 2001; Grønskov et al., 2013; Wei et al., 2013; Montoliu et al., 2014; Morice-Picard et al., 2014). The OCA5 locus is at 4q24, but the causative gene has not yet been identified (Kausar et al., 2013).

Melanin has a role in skin photoprotection against injury caused by ultraviolet radiation. Therefore, individuals affected by OCA are more susceptible to skin cancer, which is the main mortality factor associated with albinism (Moreira et al., 2013; Urtatiz et al., 2014; Yasumizu et al., 2015). Squamous cell carcinoma is the most common cancer in OCA (Graziosi et al., 2014). Incorrect melanin biosynthesis affects the visual system. Ophthalmologic characteristics of albinism include decreased visual acuity (VA), iris transillumination, nystagmus, foveal hypoplasia, misrouting of nerves fibers at the optic chiasma, photophobia, strabismus, and refractive errors (Kirkwood, 2009; Williams, 2018). Visual problems influence the quality of life of individuals with albinism by hindering normal daily activities such as watching television or reading (Omar and Fantin, 2008).

Although the different subtypes of OCA are related to mutations in different genes, clinical manifestation are often indistinguishable. Thus, molecular analysis has become an essential tool for accurate OCA subtype diagnosis, which facilitates genetic counseling and the development of new therapies (Ko et al., 2012; Summers et al., 2014; Shahzad et al., 2017; Marti et al., 2018; Onojafe et al., 2018; Lee et al., 2019). In the present study, we analyzed the spectrum of genetic mutations related to albinism in eight Brazilian patients with clinical diagnoses of OCA. To the best of our knowledge, this is the first reported genetic analysis associated with dermatologic and ophthalmologic evaluations in a cohort of pediatric Brazilian patients with OCA.

## Methods

### Participants

Eight patients with non-syndromic OCA, from seven unrelated families (siblings: C6 and C7), who attended Pequeno Príncipe Hospital were included in this study (Table 1). They were previously diagnosed with OCA by ophthalmological manifestations and hair and skin hypopigmentation without others clinical manifestations. The mean age at first evaluation was 8.8 years (minimum 1.5 years, maximum 18 years). The degree of eye, hair, and skin hypopigmentation varied between patients. Written consent was provided by their guardians. No participants were born to consanguineous parents. Concerning familial segregation mothers of participants C2, C4, C6, and C7, and mother and father of C1 and C3 agreed to participate in the study. This study was approved by the ethics committee of Faculdades Pequeno Príncipe.

**TABLE 1:** Ocular phenotypes of individuals with albinism.

**Case ID**	**A1 (years)**	**A2(years)**	**Iris translucency**	**Macular transparency**	**Nystagmus**	**Foveal hypoplasia (OCT**)	**Retinography**
C1	5	7	II	II	Yes	NA	NA
C2	1.5	3	III	I	Yes	NA	NA
C3	2	4	II	I	Yes	NA	NA
C4	2	4	III	I	Yes	NA	NA
C5	12	14	II	II	Yes	Yes	Hypopigmented
C6	14	16	II	I	Yes	Yes	Hypopigmented
C7	16	18	II	I	Yes	Yes	Hypopigmented
C8	18	20	II	II	Yes	Yes	Hypopigmented

**Case ID**	**Refraction error**	**Strabismus**		**VA1**		**VA2**	
		**E1**	**E2**	**RE**	**LE**	**RE**	**LE**

C1	HA	No	Yes	FF	FF	20/400	20/400
C2	HA	No	No	FF	FF	FF	FF
C3	H	No	No	FF	FF	20/400	20/200
C4	HA	Yes	Yes	FF	FF	20/100	20/100
C5	HA	No	No	20/400	20/400	20/400	20/400
C6	MA	Yes	Yes	20/300	20/300	20/200	20/200
C7	HA	No	No	20/200	20/200	20/200	20/200
C8	HA*	No	No	20/200	20/200	20/300	20/300

A1, age in the first ophthalmology evaluation; A2, age in the second ophthalmology evaluation; NA, not available; E1, first ophthalmology evaluation; E2, second ophthalmology evaluation; HA, hypermetropic astigmatism; H, hypermetripia; MA, myopic astigmatism; VA1, first visual acuity evaluation; VA2, second visual acuity evaluation; FF, fix and follow; RE, right eye; LE, left eye. *Refractive error shows in second evaluation. Iris translucency varies between I and III, the last being the most severe. For macular transparency, grade I is the most severe and grade III is the less transparent.

### Phenotypic Characterization

Two ophthalmic evaluations were performed for each individual, 2 years apart by the same board-certified ophthalmologist. For the first examination, visual acuity (VA) was measured using the Snellen chart in older patients (C5, C6, C7, and C8). In younger patients (C1, C2, C3, and C4), VA was measured by the patients’s ability to fix the eye and follow lights and objects. For the second assessment, the Snellen chart was used for all patients. The presence or absence of nystagmus was observed and recorded. Fundus examination was performed by direct and indirect ophthalmoscopy, and iris translucency was assessed by slit lamp examination. The results were graded according to Summers classification (Summers et al., 1988). Stereoscopic vision was measured using the Lang-Stereotest I. Optical coherence tomography (OCT) and retinography were obtained for the four older patients only. Refractive error was measured by phoropter or by retinoscopy and the strabismus was evaluated by Krimsky method.

Dermatologic evaluations were performed at the second clinical assessment for each participant and included an interview about skincare routine. Skin inspection to observe nevis and precancerous lesions was performed by a dermatologist in a room with adequate lighting. Dermatoscope Dermlite Hybrid (3 GEN LLC, San Juan Capistrano, CA, United States) DL 200 with 10× magnification was used for skin inspection.

### DNA Analysis

QIAamp DNA Blood Mini kit (Qiagen, Hilden, Germany) was used to extract DNA from the peripheral blood of all patients and five parents.

Six patients were evaluated by whole exome sequencing (WES). Exome libraries were prepared with Ion AmpliSeq^TM^ Exome RDY (Thermo Fisher Scientific, United States) following the manufacturer’s protocol. Sequencing was done using the Ion PI^TM^ Hi-Q^TM^ Sequencing 200 Kit (Thermo Fisher Scientific, United States), using Ion PI^TM^ Chip v3 (Thermo Fisher Scientific, United States). Raw data alignment with the reference genome hg19 and variant calling were performed using Torrent Suite^TM^ software (Thermo Fisher Scientific, United States). Data provided by IonReporter^TM^ platform was visualized using the Integrative Genomics Viewer software (Broad Institute, United States).

Variant selection was started by excluding the ones with minor allele frequency (MAF) >0.01. Missense, nonsense, indel and rare synonymous variants, all with MAF <0.01, were considered for further evaluation if located in genes already associated with OCA and OA or in genes listed as candidates for albinism in the literature (Kondo and Hearing, 2011; Chang, 2012) (Supplementary Table S1). Functional effects of novel potential pathogenic variations were predicted by Sorting Intolerant From Tolerant indels (SIFT_indels) and Mutation Taster in cases of indel variants. Human Splicing Finder (HSP) 3.1 was used for splicing site variants. Combined Annotation Dependent Depletion (CADD) score was calculated for the splicing and indel variants considering the threshold proposed by Mutation Significance Cutoff (MSC) for the *TYR* gene (Desmet et al., 2009; Sim et al., 2012; Schwarz et al., 2014; Itan et al., 2016; Rentzsch et al., 2019). Novel variants were named according to the Human Genomic Variation Society’s recommendations^1^ (den Dunnen et al., 2016).

Sanger sequencing was used to (a) confirm the occurrence of potential pathogenic variants found by WES, (b) conduct familial segregation studies, and (c) sequence the *TYR* gene for C5 and C6. To amplify exon 4 and 5 of the *TYR* gene, we used primers described by Chaki et al. (2005) to avoid co-amplification of the *TYRL* pseudogene (Chaki et al., 2005). Primers and full PCR amplification protocols are available upon request. Amplification products were purified with Wizard SV Gel and PCR Clean-Up System (Promega, Madison, WI, United States) amplified using BigDye^TM^ Terminator v3.1 Cycle Sequencing (Applied Biosystems, CA, United States), and direct sequenced by ABI 3500XL (Applied Biosystems, CA, United States) following the manufacturer’s protocol.

### Ancestry Analysis

The ancestries of the individuals in this study were inferred using the Peddy software package. Peddy’s ancestry classifier was modeled on the five predefined superpopulations of the 1000 Genomes Project samples, along with their genotypes at selected sites (Pedersen and Quinlan, 2017). A dimensionality reduction on the genotype matrix was conducted using principal component analysis (PCA) for each of the 1000 Genomes Project superpopulations. The resulting reduced matrix was used together with support vector machine (SVM) to build a classifier. The current study’s participants’ variant call format (VCF) files were sampled in those same sites and the fam file was obtained through Plink 1.9 (Chang et al., 2015).

## Results

### Phenotypic Characterization

All patients had iris translucency classified between II and III degrees and macular transparency between I and II degrees (Table 1). These characteristics did not change over the 2-year follow-up period. Nystagmus was present in all patients in this study (Table 1). Reduced VA were observed in all patients, except C3 who had VA measure only by her ability to fix the eye and follow lights and objects. At four patients (C5, C6, C7, and C8) who had VA measure at both evaluations, mild changes were observed in two of them (Table 1).

No patients had the ability to visualize images on the Lang-Stereotest I. At the first evaluation two patients (C4 and C6) presented with strabismus. One patient (C1) only manifested strabismus at the second evaluation (Table 1). Nystagmus hinder OCT and retinography, therefore these exams were only done in four patients (C5, C6, C7, and C8). Optical coherence tomography revealed foveal hypoplasia in all four patients. Retinography showed hypopigmented retinal pigment epithelium allowing visualization of the choroid vessels also for all four patients (Table 1).

At the first evaluation, hypermetropy was observed in two patients (C3 and C8) and astigmatism in six patients. One individual (C8) had no astigmatism at the first evaluation but developed the condition by the second evaluation (Table 1). At first evaluation, the mean spherical error in all participants was 4.20 ± 2.97 diopters, and for the second evaluation was 3.85 ± 2.71 diopters, with an average decrease of 0.35 spherical diopters between first and second evaluations. The mean astigmatism at the first evaluation was 1.73 ± 1.92 diopters and in the second evaluation the mean was 1.60 ± 1.64 diopters, with a variation of 0.13 cylindrical diopters between the two evaluations.

Of the six patients who underwent dermatological evaluation, only one individual (C3) had regular appointments with a dermatologist, and only two (C3 and C4) used sunscreen according to the recommendations of the Brazilian Society of Dermatology (Schalka et al., 2014), prior to participation in this study. All mothers reported being aware of the importance of their children using sunscreen correctly. Five of the six patients (C1, C2, C3, C6, and C7) had increasing hair and iris pigmentation since birth. Two patients (C6 and C7), who are adolescents (16 years old and 18 years old), reported being aware about the risk of skin cancer, but indicated that they do not regularly use sunscreen. One individual (C7) reported two episodes of sunstroke. During clinical evaluation, all patients and their guardians received information about sunscreen usage and the consequences of sunlight exposure, including sunburn and sunstroke, as well as skin cancer. Dermatologic examinations revealed no precancerous skin lesions in any participants.

### Genetic Analysis

Using combined WES and Sanger techniques, we identified six pathogenic variations in the *TYR* gene (C2, C5, C6, and C7) and two in the *SLC45A2* gene (C3) (Supplementary Table S2).

We identified three compound heterozygous individuals (C2, C6, and C7), and one individual (C5) who was homozygous for *TYR* gene variations. Thus, the diagnosis of OCA1 subtype was confirmed in those individuals. We identified a single heterozygosis variation (c.140G > A) in the *TYR* gene in one individual (C4), but a second causative mutation was not detected. Considering the hypothesis of a digenic form of inheritance for OCA, we also checked mutations in genes causatively linked with OCA for this individual, but we did not find a rare variant in a second gene. The only individual in this study with a molecular diagnosis of OCA4 (C3) was found to be compound heterozygous for *SLC45A2* (c.264delC/c.606G > C). No pathogenic mutation in albinism-related genes were identified in two patients (C1 and C8; Table 2). Familial segregation was confirmed for five mutations in our cohort (Table 2).

**TABLE 2:** Mutations detected in *TYR* and *SLC45A2* genes in Brazilian individuals with OCA.

**Case ID**	**Gene**	**Nucleotide changes**	**Amino acid changes**	M	**F**	***TYR* SNPs**	**OCA subtype**
C1	NId	NId	NId	−	−	R402Q	NId
C2	*TYR*	c.1217C > T	p.Pro406Leu	+	NP	S192Y	OCA 1
		**c.1185-2A > G**	ND	−	NP		
C3	*SLC45A2*	c.264delC	p.Gly89Aspfs*24	+	−	S192Y	OCA 4
		c.606G > C	p.Trp202Cys	−	+		
C4	*TYR*	c.140G > A	p.Gly47Asp	+	NP	NId	NId
		NId	NId				
C5	*TYR*	**c.1456delG**	p.Ala486Profs*11	NP	NP	NId	OCA1
		**c.1456delG**	p.Ala486Profs*11	NP	NP		
C6	*TYR*	**c.389_391delAGA**	**p.Lys131del**	−	NP	NId	OCA 1
		c.1037-7T > A	ND	+	NP		
C7	*TYR*	**c.389_391delAGA**	**p.Lys131del**	–	NP	NId	OCA 1
		c.1037-7T > A	ND	+	NP		
C8	NId	NId	NId	NP	NP	NId	NId

*NId, not identified; ND, no data; M, mother; F, father; in bold: novel mutations. (−) sequenced, mutation not identified, (+) sequenced, mutation identified, (NP) did not participate in the study. The TYR and SLC45A2 transcripts are NM_000372.5 and NM_016180.5, respectively.*

Two SNPs in the *TYR* gene, R402Q (rs1126809) and S192Y (rs1042602), which are frequently reported in association with albinism, were identified in three individuals. One patient (C1) was found to carry one copy of the R402Q variation, but no other mutations were detected. Another individual (C2) was found to carry one copy of S192Y as well as two other pathogenic variations in the *TYR* gene (c.1217C > T/c.1185-2A > G). A third individual (C3) was found to carry two pathogenic variations in *SLC45A2* (c.264delC/c.606G > C), besides S192Y, in heterozygosis (Table 2).

We found a discrepancy between WES and Sanger sequencing in one individual (C2): WES identified c.1185-2A > G in homozygosis and could not amplify part of exon 4 in the *TYR* gene. However, Sanger sequencing showed c.1185-2A > G in heterozygosis, as well as a second mutation, c.1217C > T, also in heterozygosis, which was located at the 3’ end of the reverse Ampliseq primer for amplicon TYR_115368.5578, and explains the allelic dropout for c.1185-2A > G.

Of the genetic variations detected in our cohort, three have not been previously reported as causing albinism. The *TYR* frameshift variation c.1453delG (p.Ala486Profs^∗^11), located in exon 5, was found in homozygosis in one individual (C5) and was not previously reported in the albinism database, dbSNP, CliniVar, or gnomAD. SIFT_indels predicted that c.1453delG is likely to be damaging; and Mutation Taster indicated that the variant is likely to be disease causing. Combined Annotation Dependent Depletion score was 25.3 above the MSC threshold for the *TYR* gene, which was 0.002. Hence, c.1456delG is considered to be a high impact variation. The c.1453delG variant affects amino acid 486 located in the transmembrane domain and produces a premature stop codon 11 amino acids downstream.

Two other variations, c.389_391delAGA and c.1185-2A > G, also identified in the *TYR* gene were reported in a population study, with low MAF (<0.01) but have not previously been related as causing albinism phenotype. The indel variation c.389_391delAGA leads to loss of the amino acid lysine at the 131 position, situated in the portion of tyrosinase that faces the lumen of melanosome. This mutation was found in heterozygosis in two individuals (C6 and C7) and the pathogenicity was predicted by Mutation Taster and SIFT_indels. Variation c.389_391delAGA was also determined to be a high impact variation by CADD with a score of 17.89. The splicing mutation c.1185-2A > G, located at a splicing acceptor point in intron 3, was founded in heterozygosis in one individual (C2). Human Splicing Finder 3.1 software indicated that this variation affects mRNA splicing and CADD score predicted it has a high impact with a score of 24.5. Genetic and clinical data are summarized in Table 3.

**TABLE 3:** Phenotype and genetic summary of a Brazilian pediatric albino cohort.

**Case ID**	**A1 (years)**	**Sex**	**Iris and macular translucency**	**Nystagmus**	**Retinography**	**Foveal hypoplasia**	**Visual acuity**	**Iris color**	**Dermatological findings**	**Genotype**	
										**Gene**	**Variants**
C1	5	M	+	Yes	NA	NA	Low	Dark blue	Light skin pigmentation, and dark blond hair	NId	NId
C2	1.5	F	+	Yes	NA	NA	Low	Blue	Skin hypopigmentation and blond hair	*TYR*	c.1217C > T (het) c.1185-2A > G (het)
C3	2	F	+	Yes	NA	NA	Low	Blue	Skin hypopigmentation and white-yellowish hair	*SLC45A2*	c.264delC (het) c.606G > C (het)
C4	2	M	+	Yes	NA	NA	Low	Light Blue	Milky skin and white hair	*TYR*	c.140G > A (het) WT
C5	12	M	+	Yes	Hypopigmented	+	Low	Light Blue	Milky skin and white hair	*TYR*	c.1456delG (hom)
C6	14	F	+	Yes	Hypopigmented	+	Low	Blue	Skin hypopigmentation and blond hair	*TYR*	c.389_391delAGA (het) c.1037-7T > A (het)
C7	16	M	+	Yes	Hypopigmented	+	Low	Blue	Skin hypopigmentation and blond hair	*TYR*	c.389_391delAGA (het) c.1037-7T > A (het)
C8	18	M	+	Yes	Hypopigmented	+	Low	Blue	Skin hypopigmentation and blond hair	NId	NId

*A1, age at first ophthalmology evaluation; NA, not available; NId, not identified; (+) represent the presence of clinical phenotype and (−) represents the absence of clinical phenotype; Het, heterozygous; Hom, homozygous.*

Ancestry was assessed using genomic information through Peddy. The individuals included in this study clustered together with markers corresponding to the Ad Mixed American superpopulation (Supplementary Figure S1). This superpopulation consists of Mexican, Colombian, Peruvian, and Puerto Rican samples.

## Discussion

This study provided a molecular diagnosis for five of eight people with albinism by identifying variants either in homozygosis or compound heterozygous for genes associated with OCA subtypes 1 and 4, including one novel causal mutation in the *TYR* gene (c.1453delG). We also report two rare variants (previously identified in a populational study) to be associated as cause of albinism, for the first time.

Translucent iris, transparent macula, and nystagmus contribute to the impairment of quality of life (Kirkwood, 2009; Sheth et al., 2013; Maia et al., 2015; McCafferty et al., 2015). As expected, all participants in the current study presented this ophthalmologic profile. Study participants were all unable to visualize 3D images on Lang-Stereotest I. This demonstrates the decreased stereoscopic vision which is typical in albinism and is caused by misrouting optic fibers on the chiasma. Reduced VA is another important manifestation of OCA clinical diagnosis. The best VA for any participant in the current study was 20/100, which demonstrates the severe reduction of VA which is common for albino individuals. No significant changes in VA occurred during the 2-year follow-up. No other study presenting an ophthalmologic follow-up was found in the literature for comparison with our cohort, specially considering the broad range of age.

Two individuals presented with strabismus at both evaluations: one (C1) had convergent strabismus; and the other (C6) had divergent strabismus. One individual (C1) presented with divergent strabismus which was only observed at the second evaluation (Table 1). Strabismus is not an important factor for the clinical diagnosis of albinism but is often associated with the condition (Williams, 2018), as demonstrated by the three patients in this study with the condition.

Refractive errors are also often associated with albinism (Kirkwood, 2009). In this study, the most frequent ametropia was astigmatism, which was observed in seven patients. Hypermetropic astigmatism was the most common refractive error found in the current study’s patients which is consistent with other reports (Yahalom et al., 2012; Schweigert et al., 2018). Overall, we observed an average decrease of 0.35 spherical diopters and 0.13 cylindrical diopters. This suggests that albinism could follow the physiological tendency of a slow and gradual decrease of spherical diopters (hypermetropia) in the first years of life and the tendency of stability of cylindrical diopters (astigmatism) in the later years of life (Marsh-Tootle and Frazier, 2006). A recent retrospective study with a group of children with albinism showed a tendency of increasing the cylindrical rate in the first 10 years of life (Schweigert et al., 2018). However, half of the individuals in our study were older than 15 years in contrast to the study by Schweigert et al. (2018), whose maximum age was 10 years, making it difficult to compare results.

Five individuals underwent one dermatological evaluation. For each of the patients included in this study, increasing hair and eye pigmentation from birth was reported by the patients’ mothers, except one. This is in agreement with the literature in connection with subtypes OCA1B through OCA7 (Grønskov and Brondum-Nielsen, 2007; Gargiulo et al., 2011; Kamaraj and Purohit, 2014). Consistent with what is observed in the general population (Fabris et al., 2012), the current study participants did not use sunscreen regularly. The lack of melanin among people with albinism causes an increased susceptibility to skin cancer, which is the mainly mortality factor for albinism. Thus, sunscreen is particularly important for skin cancer prevention in this group (Kromberg et al., 1989; Moreira et al., 2013; Kiprono et al., 2014; Yasumizu et al., 2015). We did not find any precancerous lesions in the study cohort. This was expected because the study participants were pediatric patients and the literature reports these lesions occur more commonly in the second decade of life in individuals with albinism (Kiprono et al., 2014).

In the current study, genetic analysis showed an association of the SNP R402Q with albinism. This has often been reported in the literature (Simeonov et al., 2013). Some studies demonstrated that this mutation results in a temperature-sensitivity protein which has reduced activity and is retained in endoplasmic reticulum at 37°C (Tripathi et al., 1992; Spritz et al., 1997; Berson et al., 2000; Halaban et al., 2000). However, the variant itself appears unable to cause albinism, because unaffected relatives homozygous for R402Q do not shown albinism traits (Oetting et al., 2009; Preising et al., 2011). We identified the R402Q in one individual (Table 2) who does not carry other variants in the *TYR* gene. S192Y is another common non-pathogenic polymorphism observed in two individuals in the current study. It has been reported that this variant reduces tyrosinase activity in 40% (Chaki et al., 2011). In the current study, one individual with S192Y SNP had two other variants, *TYR* and *SLC45A2*, which could explain their albino phenotype (Table 2).

As larger groups of people with albinism are studied, new variants in known genes are being reported. A recent study showed a cohort of 990 patients and introduced 245 novel mutations in genes already correlated to albinism. However, researchers were unable to conclude the molecular diagnosis for approximately 27% of participants (Lasseaux et al., 2018). Another study reported 31 new mutations in a cohort of 114 individuals with albinism and also reported 14% participants without identified mutations (Zhong et al., 2019). Despite a smaller group of subjects, our study identified one novel mutation and two rare variants, as cause of OCA.

**FIGURE 1:**
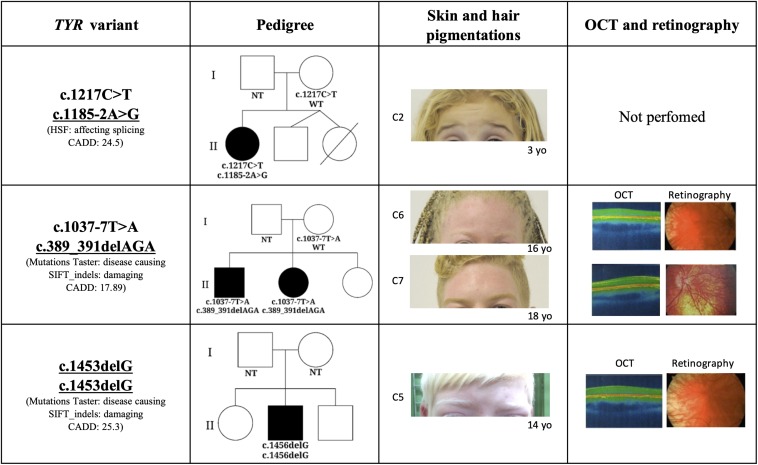
Phenotypic characteristics associated with novel mutations in *TYR* gene. The threshold of *TYR* CADD score is 0.002. C2: Case 2; C6: Case 6; C7: Case 7; OCT: Optical coherence tomography; NT, not tested; WT, wild type; HSF, Human Splicing Finder; and CADD, Combined Annotation Dependent Depletion.

A novel frameshift *TYR* variant, c.1453delG, was identified in homozygosis in one patient which was consistent with the autosomal recessive pattern of inheritance for OCA. The patient’s skin color, hair color, and ophthalmologic characterization (foveal hypoplasia, nystagmus, reduced VA, and translucent iris) were consistent with the albino phenotype (Figure 1). *In silico* analysis predicted this variant to be damaging (Mutation Taster) and disease causing (SIFT_indels). Combined Annotation Dependent Depletion also assumed c.1453delG to be a high impact variant. As the variant results in a shorter tyrosinase and affects the transmembrane domain, we hypothesize that not anchoring to the melanosome membrane may prevent tyrosinase from functioning, giving rise to the clinical phenotype observed in Case 5.

Two other mutations identified in the *TYR* gene (c.1185-2G > A and c.389_391delAGA) were previously described in a *Trans*-Omics for Precision Medicine (TOPMed) study. The TOPMed study, which was part of a broader precision medicine initiative aiming to provide treatment for genetic disease caused by one particular gene, performed whole-genome sequencing in 144 thousand patients across more than 80 different studies. Neither mutation was previously connected with a specific phenotype and their pathogenesis had not yet been predicted. The TOPMed study identified the indel mutation c.389_391delAGA in the freeze 5 panel which was composed from individuals with European, African, Hispanic/Latino, and East Asian ancestry. This mutation was reported in heterozygosis, in two individuals with African ancestry. c.389_391delAGA is located in the exon 3 and causes loss of lysine in position 131 and it is a conserved amino acid in the evolutionary scale of mammals. Mutation Taster, SIFT_indels, and CADD predicted the potential pathogenesis of the c.389_391delAGA mutation to be damaging, disease causing and have a high impact on gene transcription. In our cohort, the variant c389_391delAGA was identified in siblings (C6 and C7) whose ancestry analysis clustered them with Ad Mixed American instead of African population, this is in agreement with the TOPMed freeze 5 panel study. A second mutation, c.1037-7T > A, which was previously reported as causing OCA1 (Park et al., 1997; Grønskov et al., 2009; Lasseaux et al., 2018; Zhong et al., 2019), segregated in trans with c.389_391delAGA. Both mutations were identified in individuals (C6 and C7) who presented ophthalmologic manifestations and hair and skin hypopigmentation consistent with the albinism phenotype (Figure 1).

The splicing variation c.1185-2G > A was also identified in the TOPMed freeze 5 panel, without disclosure of ancestry. Analysis of HSP 3.1 concluded that the c.1185-2G > A is located in the acceptor splicing site of the *TYR* gene, affecting mRNA splicing. Here too CADD score indicated that this variant probably has a high impact on the protein. The c.1185-2G > A variation may result in skipping of the entire exon 4 which codes the region close to the second Cu binding site of tyrosinase. The splicing mutation in trans with c.1217C > T was identified in one patient (C2; Table 2) who presented with the typical albinism phenotype (light blond hair, pale skin, light blue eyes, translucid iris, macular transparency, nystagmus, and reduced AV; Figure 1).

We report three novel mutations causing albinism. For three individuals with the classic OCA phenotype, we could not conclude molecular diagnosis. In OCA, a lack of associated genes or only one mutant allele in patients with clinical diagnosis of OCA has been frequently reported in the literature (Grønskov et al., 2009; Gargiulo et al., 2011; Montoliu et al., 2014; Lasseaux et al., 2018; Zhong et al., 2019). Pathogenic variations may be in intragenic, intronic, or regulatory regions that were not covered by the techniques we used. Intragenic rearrangements, which could also explain these cases, were not evaluated. In addition, causative mutations may also be located in other genes not yet associated with OCA.

Efforts for the development of studies with large case series, including age matched groups, should be considered since a small group of individuals with a broad range of age imposes limitations for clinical follow up conclusions. Genetic studies with these large cases series, diverse populations, and new molecular approaches to define genetic etiology may further the development of precision medicine for conditions affecting melanogenesis.

## Data Availability Statement

The datasets generated for this study can be found in ClinVar, accession number SCV000925975.1 (c.1456delG), SCV001132036 (c.1185-2A>G/rs1289685376) and SCV000998903 (c.389_391delAGA/rs1413017181).

## Ethics Statement

The studies involving human participants were reviewed and approved by Comitê de Ética em Pesquisa, Faculdades Pequeno Príncipe, Curitiba, Brazil. Written informed consent to participate in this study was provided by the participants’ legal guardian/next of kin. Written informed consent was obtained from the minor(s)’ legal guardian/next of kin for the publication of any potentially identifiable images or data included in this article.

## Author Contributions

LS performed WES and Sanger sequencing reactions and data analysis and wrote draft of the manuscript. RR performed WES reactions. AJ, YW, and YI performed ancestry analysis. RR and CP contributed to sequencing data analysis. FL and PI performed ophthalmologic evaluation. LS, FL, and CP reviewed patients’ charts. PR, RAR, and NA were responsible for dermatological evaluation. LS and CP wrote sections of the manuscript. CP designed the study. All authors contributed to manuscript revision, read and approved the submitted version.

## Conflict of Interest

The authors declare that the research was conducted in the absence of any commercial or financial relationships that could be construed as a potential conflict of interest.

## Footnotes

1 http://varnomen.hgvs.org/
