# Supplementary material for: Non-syndromic Oculocutaneous Albinism: Novel Genetic Variants and Clinical Follow Up of a Brazilian Pediatric Cohort
Source: Front Genet. 2020 Apr 28;11:397. doi: 10.3389/fgene.2020.00397 (PMC7198815; doi:10.3389/fgene.2020.00397)
Supplement: Supplementary file 1 [file Data_Sheet_1.docx]

**Supplementary Table 1 Albinism genes and candidate genes analyzed in the exome sequencing.**

|  | **Analyzed Gene** | **Location** | **Size (pb)** | **Exons** | **Protein (aa)** | **RefSeq** |
| --- | --- | --- | --- | --- | --- | --- |
| ***Albinism related genes*** | *TYR* | 11q14.3 | 2485 | 5 | 529 | NM_000372.5 |
|  | *OCA2* | 15q12-q13 | 3186 | 24 | 838 | NM_000275 |
|  | *TYRP1* | 9p23 | 2851 | 8 | 537 | NM_000550 |
|  | *SLC45A2* | 5p13.2 | 1772 | 7 | 530 | NM_016180.5 |
|  | N.I. | 4q24 | N.A. | N.A. | N.A. | N.A. |
|  | *SLC24A5* | 15q21.1 | 1897 | 9 | 500 | NM_205850 |
|  | *LRMDA* | 10q22.2-q22.3 | 1040 | 7 | 226 | NM_001305581 |
|  | *GPR143* | Xp22.2 | 1762 | 9 | 404 | NM_000273 |
| ***Albinism candidate genes*** | *DCT* | 13q32.1 | 5074 | 8 | 519 | NM_001922.5 |
|  | *MITF* | 3p13 | 4670 | 10 | 504 | NM_198177.3 |
|  | *MC1R* | 16q24.3 | 3099 | 1 | 317 | NM_002386.3 |
|  | *POMC* | 2p23.3 | 1128 | 3 | 267 | NM_000939.4 |
|  | *ASIP* | 20q11.22 | 586 | 3 | 132 | NM_001672.2 |
|  | *SLC24A4* | 14q32.12 | 4531  N.I: not identified; N.A: not available. | 18 | 603 | NM_153647.4 |
|  | *IRF4* | 6p25.3 | 5314 | 9 | 451 | NM_002460.4 |
|  | *TPCN2* | 11q13.3 | 4969 | 25 | 752 | NM_139075.4 |

**Supplementary Table 2 Oculocutaneous Albinism related variants found in the cohort of eight Brazilian individuals.**

(+) represent that the variant has been related to albinism, (-) present that the variant has not been related to albinism before the present study; N.Av.: not available; N.Ap: does not apply.

| **Variant** | **Gene** | **Type of mutation** | **Associated with Albinism** | **dbSNP** | **Allele Frequency in dbSNP** | | | |  | **Pathogenicity Predictions** | | | |
| --- | --- | --- | --- | --- | --- | --- | --- | --- | --- | --- | --- | --- | --- |
|  |  |  |  |  | **gnomAD** | **ExAC** | **1000G** | **TOPMED** |  | **SIFT_indel** | **Mutation Taster** | **HSP 3.1** | **CADD score** |
| c.1217C>T | *TYR* | missense | + | rs104894313 | T=0.00383 | T=0.00349 | T=0.002 | T=0.00349 |  | N.Ap. | Disease causing | N.Ap. | 32.0 |
| c.1185-2A>G | *TYR* | splicing | - | rs1289685376 | N.Av. | N.Av. | N.Av. | G=0.00001 |  | N.Ap. | N.Ap. | Affecting Splicing | 24.5 |
| c.140G>A | *TYR* | missense | + | rs61753180 | A=0.00016 | A=0.00009 | A=0.001 | A=0.00026 |  | N.Ap. | Disease causing | N.Ap. | 27.3 |
| c.1456delG | *TYR* | frameshift deletion | - | N.Av. | N.Av. | N.Av. | N.Av. | N.Av. |  | Damaging | Disease causing | N.Ap. | 25.3 |
| c.389_391delAGA | *TYR* | inframe deletion | - | rs1413017181 | delAGA=0.0001 | N.Av. | N.A. | delAGA=0.00001 |  | Damaging | Disease causing | N.Ap. | 17.89 |
| c.1037-7T>A | *TYR* | splicing | + | rs61754381 | A=0.0002 | N.Av. | A=0.001 | A=0.00042 |  | N.Ap. | N.Ap. | Affecting Splicing | 5.06 |
| c.264delG | *SLC45A2* | frameshift deletion | + | rs775387808 | delG=0.00010 | delG=0.00008 | N.Av. | delG=0.00014 |  | Damaging | Disease causing | N.Ap. | N.Av. |
| c.606G>C | *SLC45A2* | missense | + | rs146802593 | G=0.00023 | G=0.0002 | N.Av. | G=0.00013 |  | N.Ap. | Disease causing | N.Ap. | 23.1 |


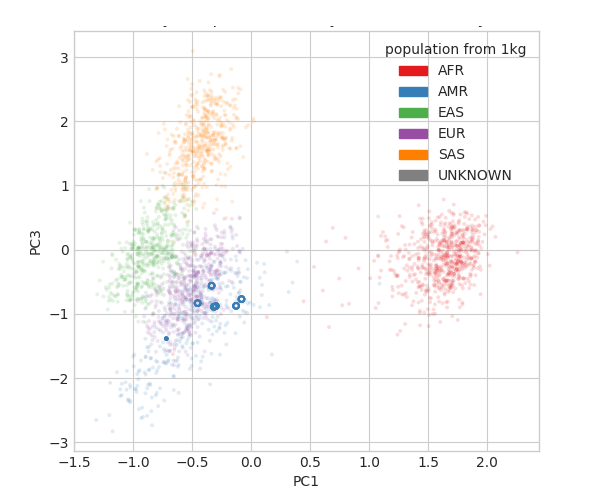


Supplementary Figure 1: Principal component analysis (PCA) of ancestrally analysis. The participants clustered together with markers corresponding to the Ad Mixed American superpopulation (dots circled in blue).
